# Storage Quality Variation of Mushrooms (*Flammulina velutipes*) after Cold Plasma Treatment

**DOI:** 10.3390/life13010070

**Published:** 2022-12-26

**Authors:** Yuxuan Ding, Weixian Mo, Zilong Deng, Benard Muinde Kimatu, Juan Gao, Donglu Fang

**Affiliations:** 1 Co-Innovation Center for Sustainable Forestry in Southern China, College of Forestry, Nanjing Forestry University, Nanjing 210037, China; 2 College of Food Science and Technology, Nanjing Agricultural University, Nanjing 210095, China; 3 State Key Laboratory Pollution Control, School of Environmental Science and Engineering, Tongji University, Shanghai 200092, China; 4 Department of Dairy and Food Science and Technology, Egerton University, Egerton 20115, Kenya

**Keywords:** cold plasma, *Flammulina velutipes*, cell membrane integrity, storage quality, antioxidant enzyme activity

## Abstract

*Flammulina velutipes* is susceptible to mechanical damage, water loss, microbial growth, and other factors that lead to postharvest deterioration, thereby shortening the storage period. The purpose of this study was to analyze the effects of cold plasma treatment on the physicochemical properties and antioxidant capacity of *F. velutipes* during storage at 4 °C for 21 days. Compared to the control group, cold plasma cold sterilization (CPCS) treatment (150 Hz, 95 kV for 150 s) effectively inhibited the growth and multiplication of microorganisms on the surface of *F. velutipes*, with no significant effect on the fresh weight change and the superoxide anion generation rate, but with a higher postharvest 1,1-dephenyl-2-picrylhydrzyl (DPPH) clearance rate. Moreover, CPCS increased antioxidant enzyme activities, delayed both malondialdehyde (MDA) accumulation and vitamin C loss, inhibited the browning reaction and polyphenol oxidases (PPO) activity and protected *F. velutipes* cell membrane from disruption. In general, CPCS not only achieved bacteriostatic effects on *F. velutipes* during storage, but also reduced cell damage from free radical oxidation, resulting in better postharvest quality and longer shelf life.

## 1. Introduction

*Flammulina velutipes*, commonly known as golden needle mushroom, has a long history of cultivation worldwide with a high degree of industrial-scale production because of its delicious taste and rich variety of active substances, including polysaccharides, streptocin, ergosterol, active proteins, and polypeptides [1]. Fresh *F. velutipes* after harvest possesses high moisture content (nearly 90%) and lacks a cuticle on the surface of mushroom cap or stem [2]. The fruiting bodies of *F. velutipes* are easily damaged and deteriorate after harvest, owing to their high respiration and metabolism rates, external microorganism attack, and other factors [3]. This short shelf life severely restricts the development of the *F. velutipes* industry.

At present, *F. velutipes* preservation research mainly adopts modified atmosphere packaging [3], 1-MCP treatment [4], and nanocomposite packaging materials [5,6], among other technologies. However, the drawbacks of these methods are complex operation, limited fresh-keeping effects, and food nutrition and safety problems. Plasma is considered to be the fourth state of matter after solid, liquid, and gas. It exists in various forms in nature and is mainly composed of electrons, ions, neutral particles, ground-state or excited-state molecules, and electromagnetic radiation [7]. In recent years, cold plasma cold sterilization (CPCS) has been considered more competitive than low-pressure plasma in industrial applications because of its technological and economic advantages [8,9], and it is available in food packaging. CPCS treatment produces reactive oxygen species (ROS), including hydroxyl radicals, hydrogen peroxide, and superoxide anions, which can affect cellular activity and damage DNA molecules, resulting in the direct oxidation of microbial cells; enzymes can also be inactivated by free radicals and atomic oxygen-mediated oxidation reactions [10]. As a result, CPCS technology can inhibit microbial growth and trigger the inactivation of endogenous enzymes and thus has been developed as a non-thermal treatment technology for applications in food [11]. The application of plasma-activated liquid (PAL), including plasma-activated water (PAW) and plasma-activated buffer solutions (PABS), to tomato fruits effectively reduces two pesticide residues, chlorothalonil (CTL) and thiram (THM), and improves food safety [12]. Cold plasma treatment of alfalfa, onion, radish, and cress seeds effectively inactivates bacteria on the seeds while maintaining their germination properties, with mild plasma treatment conditions promoting alfalfa germination [13]. Compared with the control group, CPCS significantly improved the germination rate of mung bean seeds and simultaneously promoted an increase in soluble sugar, protein, and hydrolase activities, which is beneficial to seed germination under drought conditions [14]. In terms of preservation of fresh fruits and vegetables after different cold plasma treatment times, sampling and testing at 1, 2, and 7 days showed that compared with the control group, the total number of blueberry colonies in the experimental group was significantly reduced after treatment for more than 60 s, which caused a significant reduction in hardness and affected anthocyanin content [15]. Tomatoes treated with an intermittent corona discharge plasma jet (ICDPJ) and stored at 25 °C for 15 days showed reduced pollutant loads and no significant differences in taste, flavor, color, and significantly longer shelf life of 10–15 days [16]. Therefore, CPCS can be used as a green technology for the preservation of fruits and vegetables. At present, most studies have focused on the sterilization effect of cold plasma; there are few studies regarding the impact on the quality of the treated products during storage, and there is a lack of information on its application in edible fungi preservation.

In this experiment, we studied the physiological and biochemical changes, antioxidant capacity, and cell microstructure of *F. velutipes* after treatment with optimum CPCS conditions. The effects of CPCS on microbial growth, storage quality, cell integrity, free radical scavenging, and antioxidant enzyme activity of *F. velutipes* during low-temperature storage were evaluated through microbial experiments, physiological and biochemical index determination, and electron microscope observation. The results provide a theoretical basis for the application of cold plasma technology in the field of *F. velutipes* preservation.

## 2. Materials and Methods

### 2.1. Sample Source and Pre-Treatment

*F. velutipes* used in this study was obtained from a commercial farm in Jiangsu, China, and transported to the laboratory within half an hour. *F. velutipes* with a complete mushroom body, white color, unopened umbrella, and no pests or mechanical injuries were selected. *F. velutipes* was then stored in cold storage at 4 °C immediately after delivery to the laboratory and pre-cooled for 24 h.

### 2.2. Cold Plasma Treatment Conditions Optimization

#### 2.2.1. Single-Factor Experiment

*F. velutipes* (50 g) was randomly packaged in a polypropylene packaging box (17 cm × 12 cm × 3.2 cm) and sealed. The processing time (60, 90, 120, 150, 180, and 210 s), the processing voltage (55, 65, 75, 85, 95, and 105 kV), and the processing frequency (60, 90, 120, 150, 180, and 200 Hz) of the dielectric barrier discharge (DBD) cold plasma were set as the optimization conditions. Air was used as the working gas, and the distance between the electrodes was 29 cm. The sample in the sealed box was then processed. Each treatment was repeated three times. *F. velutipes* without plasma treatment was used as the control. The microbial indicator (total number of microbial colonies) of *F. velutipes* was measured three times in each group. Detailed information regarding the single-factor experiment is provided in Supplementary Figure S1.

#### 2.2.2. Response Surface Method Optimization

Based on the single-factor test results, taking the treatment voltage, treatment frequency, and treatment time as independent variables and the sterilization rate as the response value, the response surface optimization of the sterilization rate of *F. velutipes* was carried out (Supplementary Tables S1 and S2).

According to the analysis from the Design Expert software, the optimal process conditions are a processing frequency of 151.631 Hz, a processing voltage of 94.308 kV, and a processing time of 150.102 s, under which the sterilization rate reaches 99.440%. Considering the requirements of the actual equipment parameter settings, the optimal process conditions were adjusted as follows: a processing frequency of 150 Hz, a processing voltage of 95 kV, a processing time of 150 s, and a corrected sterilization rate of 99.439% (n = 3).

### 2.3. Sample Preparation, Treatment, and Storage

Comparative experiments of CPCS in mushroom preservation were then conducted to optimize the CPCS parameters. *F. velutipes* (50 g) was randomly packaged in polypropylene packaging boxes (17 cm × 12 cm × 3.2 cm) and sealed. The processing time of the dielectric barrier discharge (DBD) cold plasma equipment was set to 150 s, the processing voltage was 95 kV, and the processing frequency was 150 Hz. The size of the electrodes was 17 cm × 13 cm. Each sealed box was used as a group for subsequent experiments. *F. velutipes* without plasma treatment was used as a control. All samples were stored at 4 °C and a 90% relative humidity for 24 days. The growth changes, appearance, and physiological and biochemical indicators of *F. velutipes* were measured every three days, and each measurement was repeated three times. The samples were removed from the sealed box during testing. Each parameter was evaluated using three boxes of *F. velutipes* at each time point.

### 2.4. Appearance Evaluation and Microbial Colony Assay of Mushrooms

#### 2.4.1. Appearance Evaluation

In order to keep track of the exterior quality changes of *F. velutipes*, a cluster of samples from each treatment were taken out from the packages and photographed every three days on a black background according to our previous study [5]. The physiological phenomenon of postharvest mushroom including browning, cap opening, and stem elongation were observed and recorded.

#### 2.4.2. Microbial Colony Assay of Mushrooms

The microbial colony assay was conducted using the standard plate count method [17]. In brief, *F. velutipes* (25 g) was chopped on an ultra-clean workbench, placed in a conical flask containing 225 mL of normal saline, shaken and homogenized for 30 s for appropriate gradient dilution. The appropriate dilution gradient (normal saline, 1 mL) was poured onto a plate that contains 15−20 mL of agar media, and each gradient was repeated three times and cultured in a 37 °C incubator for 48 ± 2 h. The sterilization rate was determined by measuring the total numbers of colonies of *F. velutipes* in the control and CPCS groups before and after the storage period. The sterilization rate was expressed as a percentage of the total number of *F. velutipes* colonies in the CPCS group relative to the total number of *F. velutipes* in the control group.

### 2.5. Physico-Chemical Analysis

#### 2.5.1. Weight Loss, Cap Opening Rate, and Elongation of the Mushroom Stem

The external quality including weight loss, the cap opening rate, and the stem elongation rate was analyzed referring to our previous experiment [18]. Weight loss was determined by weighing the whole mushrooms before and after storage. Weight loss was expressed as a percentage of the weight with respect to the initial weight. The equation is shown below:

$$Weight\;loss\left(\%\right)=\frac{M_{0}-M_{n}}{M_{0}}\times 100$$

where *M*_0_ is the initial weight of the *F. filiformis* and *M_n_* is the weight of *F. filiformis* on each sampling day.

The cap opening rate was described as a percentage of open caps with the following equation:

$$Cap\;opening\;rate\left(\%\right)=\frac{N_{oc}}{N_{t}}\times 100$$

where *N_oc_* is the number of *F. filiformis* with open caps on each sampling day and *N_t_* is the total number of mushrooms.

The stem elongation rate was expressed as the percentage of mushroom stem elongation and calculated using the following equation:

$$Stem\;elongation\;rate\left(\%\right)=\frac{E_{n}-E_{0}}{E_{0}}\times 100$$

where *E*_0_ is the initial *F. filiformis* stem length and *E_n_* is the *F. filiformis* stem length on each sampling day.

#### 2.5.2. Total Phenolic Content, Soluble Solids Content, and Vitamin C

The total phenolic content was tested according to the method of Bristy [19]. In brief, chopped *F. velutipes* (2.0 g) was added to a 1% (*v*/*v*) HCl−methanol solution and ground into a homogenate in an ice bath. The mortar was washed with 1% HCl−methanol solutions, and the contents were transferred to a 20 mL flask. With a 1% HCl−methanol solution, the contents were made to volume, mixed well and extracted at 4 °C in the dark for 30 min. The mixture was centrifuged at 10,000 rpm for 20 min, and the absorbance of the supernatant was measured at 760 nm using a 1% HCl−methanol solution as a control. Gallic acid was used to construct a standard curve to calculate the total phenolic mass fraction (μg/g) in the samples.

To determine the soluble solids content, *F. velutipes* (1.0 g) was fully ground. Next, 5 mL of deionized water were added and mixed well, and then samples were filtered. The supernatant was used for soluble solid content measurement at 25 °C using a sugar content meter (PAL-1, Japan Aituo Co., Ltd., Tokyo, Japan) [20]. The results were expressed as percentages (%).

The vitamin C content was analyzed referring to Ghosh’s method [21]. *F. velutipes* (2.0 g) was added to 5 mL of an oxalic acid−EDTA solution and ground into a homogenate in an ice bath. The mixture was then centrifuged at 6000 rpm for 15 min at 4 °C. Then, 2 mL of supernatant were mixed with 3 mL of a 0.05 mol/L oxalic acid−EDTA solution, 0.5 mL of a 1 mg/mL metaphosphoric acid−acetic acid solution, 1.0 mL of a 5% sulfuric acid solution, and 2.0 mL of a 5% ammonium molybdate solution. The mixture was diluted to 20 mL with distilled water and then incubated in a water bath at 80 °C for 1 h before measuring absorbance at 760 nm using a UV-visible spectrophotometer (Alpha-1860A, Shanghai Puyuan Co., Ltd., Shanghai, China). Vitamin C was used as the standard curve to calculate the vitamin mass fraction (μg/g) of the sample.

#### 2.5.3. Malondialdehyde Content and Browning Degree

Malondialdehyde (MDA) content was determined using a lipid hydrogen peroxide assay kit (Beijing Solarbio Technology Co., Ltd., Beijing, China).

To determine the browning degree, *F. velutipes* (2.0 g) was ground thoroughly, 5 mL of a sodium phosphate buffer (pH = 6.5, 0.2 mol/L) were added, and the mixture was allowed to stand for 10 min before being centrifuged (4 °C, 8000 r/min, 15 min). The absorbance of the supernatant was measured at 450 nm and expressed as OD_450_ × 2.

#### 2.5.4. Antioxidant Enzyme Activity

Superoxide dismutase (SOD) enzyme activity was determined using a superoxide dismutase detection kit (Beijing Boxbio Science and Technology Co., Ltd., Beijing, China). Peroxidase (POD) enzyme activity was determined using a peroxidase activity assay kit (Beijing Solarbio Technology Co., Ltd., Beijing, China). Catalase (CAT) enzyme activity was determined using a catalase detection kit (Shanghai Yuanye Biotechnology Co., Ltd., Shanghai, China). Polyphenol oxidase (PPO) enzyme activity was determined using a polyphenol oxidase activity assay kit (Beijing Solarbio Technology Co., Ltd., Beijing, China). All measurements were performed according to the manufacturer’s instructions.

#### 2.5.5. Free Radical Scavenging Ability

The rate of superoxide anion generation in *F. velutipes* was determined using a superoxide anion content detection kit (Beijing Boxbio Science and Technology Co., Ltd., Beijing, China).

Two grams of *F. velutipes* were added to 5 mL of pre-cooled anhydrous ethanol, and the mixture was ground in an ice bath. The mixture was centrifuged at low temperature (4 °C, 10,000× *g*, 20 min). Then, 0.3 mL of extract was mixed with distilled water (9.0 mL) and vortexed. A 2 mL volume of this diluent was mixed with an equal volume of a 0.1 mmol/L 1,1-dephenyl-2-picrylhydrzyl (DPPH) ethanol solution. The mixture was incubated at 20−25 °C for 30 min, and the absorbance value A_0_ of the control group at 517 nm and the absorbance value A_1_ of the sample at 517 nm were measured.

The DPPH free radical scavenging rate (%) was calculated as [(A_0_−A_1_)/A_0_] ×100.

### 2.6. Transmission Electron Microscope (TEM) Observation

*F. velutipes* samples stored for 0, 12, and 24 days were placed in a pre-cooled 4% glutaraldehyde solution, cut into thin strips of a 2−5 mm length, a 1−2 mm width, and a 1 mm thickness, transferred into a pre-cooled glutaraldehyde fixative in a 2 mL centrifuge tube and fixed at 4 °C for more than 8 h. After the samples were rinsed, dehydrated, replaced, embedded, ultrathin sectioned and stained, they were observed using a transmission electron microscope (7800, HITACHI, Ibaraki, Japan).

### 2.7. Statistical Analyses

All indicators were measured in triplicates. The data are expressed as mean ± SD, and the experimental data were organized and plotted using Excel software. Analysis of variance (ANOVA) was performed using SAS 9.4 software. To determine the statistical differences, comparisons of the means between the control and treatment samples were performed using Duncan’s test at a significance level of *p* < 0.05.

## 3. Results

### 3.1. Physical Structure Evaluation

**Figure 1:**
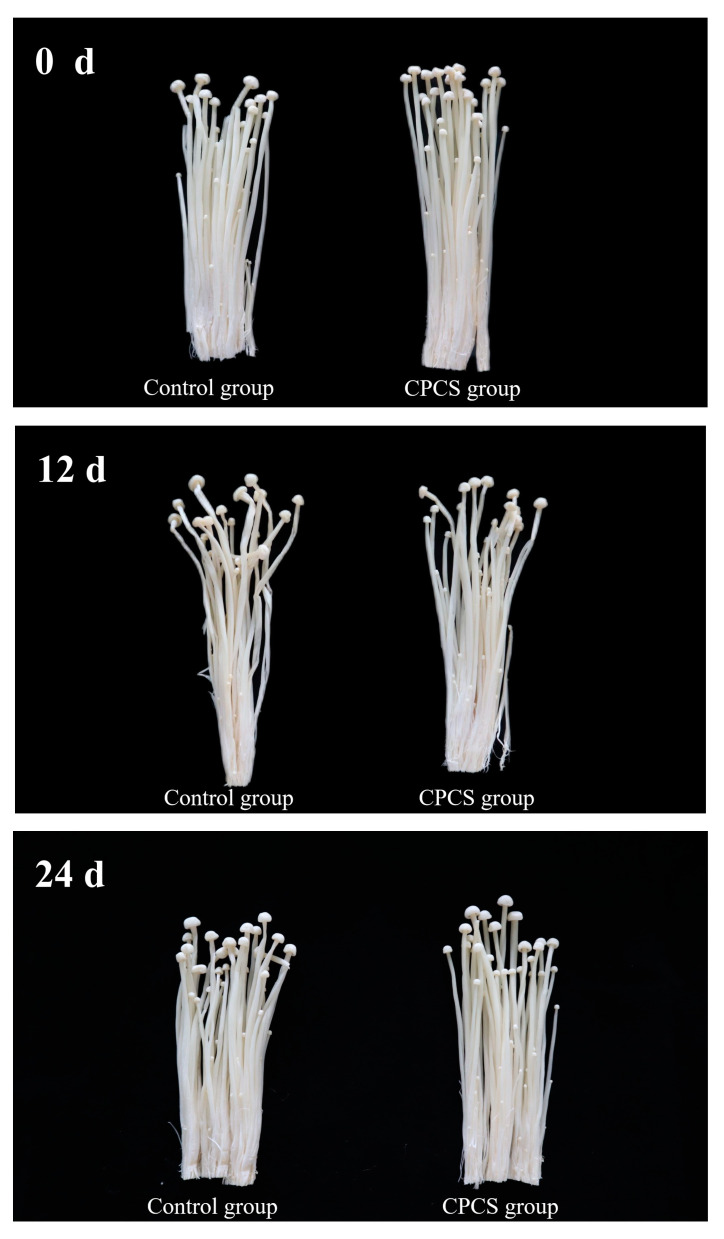
Effects of CPCS on the sensory quality of *F. velutipes* during cold storage.

Untreated and CPCS-treated *F. velutipes* were evaluated every three days during the 24-day storage period. As shown in Figure 1, after the control and CPCS groups were stored, a certain amount of water appeared in the packaging box and the color changed from milky white to milky yellow. In the later stage of storage, the control group showed slight browning, and the caps appeared shriveled to a certain extent. Compared to the change in the color of *F. velutipes* after 10 days of storage following 1-MCP treatment [4], there was less change after CPCS treatment.

### 3.2. The Total Number of Colonies

With prolonged storage, the total microbial number of *F. velutipes* gradually increased (Table 1). From 12 to 15 days, the microbial growth rate of the control group was the fastest, at 489.6%, while the total number of colonies in the CPCS group was significantly lower than that of control group during storage (*p* < 0.05) and effectively inhibited microbial growth.

### 3.3. Weight Loss, Cap Opening Rate, and Elongation of the Mushroom Stem

**Figure 2:**
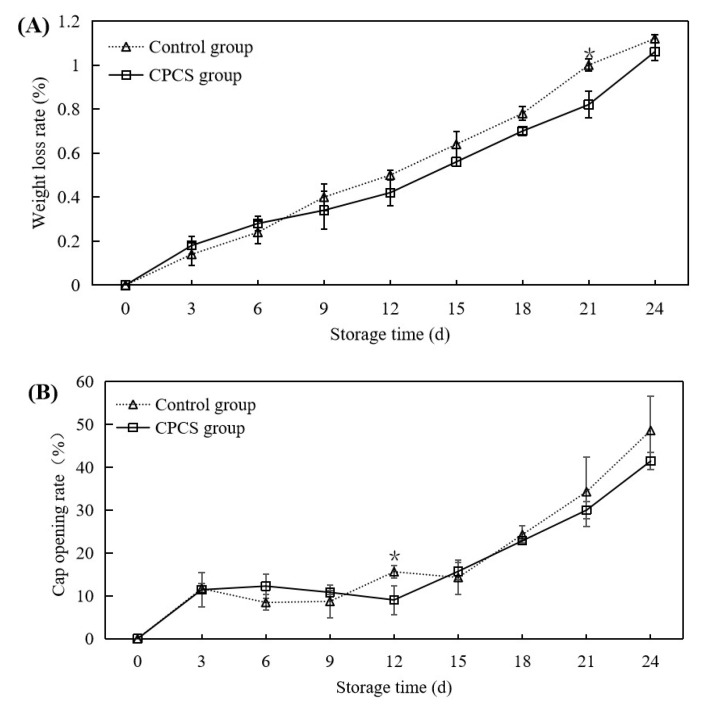

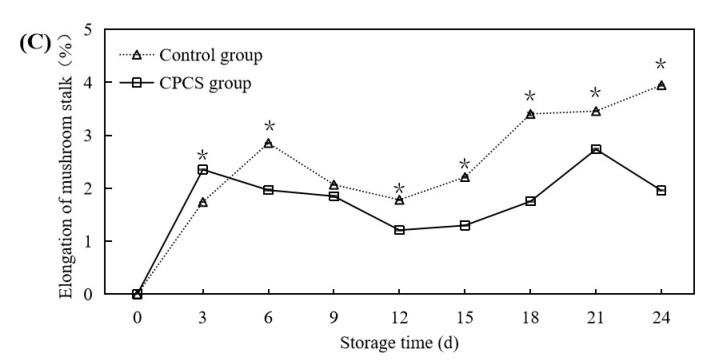
Effects of CPCS on weight loss (**A**), the cap opening rate (**B**), and stem elongation (**C**) of *F. velutipes* during cold storage. ‘*’ indicated the significant differences (*p* < 0.05) between two treatments on each sampling day.

Postharvest maturation of *F. velutipes* occurs mainly in the form of stem elongation, cap opening, fibrillation, riverside, and spore formation. These phenomena are a manifestation of the aging of *F. velutipes* and result in its reduced commercial value [22]. The weight loss rate, the cap opening rate, and stem elongation are important indicators that affect the quality and commercial value of *F. velutipes*: Compared to that of the control, the weight loss rate of the CPCS group was not significantly different on the 3rd and 6th days of storage, whereas for the rest of the storage period, the measurements were remarkably lower than those of the control group (Figure 2A). At the later stage of storage, the fresh weight of the control group was 49.44 ± 0.18 g, and the fresh weight of the experimental group was 49.47 ± 0.22 g. There was no significant difference in fresh weight between the two groups (*p* > 0.05). However, the cap opening rates of *F. velutipes* between the control and CPCS groups were significantly different only on the 12th day of storage (*p* < 0.05). At the end of storage, the cap opening rate of *F. velutipes* in the control group reached 48.57%, which was 7.15% higher than that of the CPCS group (Figure 2B). Since the stems of *F. velutipes* continued to elongate after harvesting, from the 6th day of storage, the stem elongation of *F. velutipes* in the control group was significantly higher than that of the CPCS group (Figure 2C). In summary, CPCS-treated *F. velutipes* slowed down the weight loss rate, opening rate, and stalk elongation during post-ripening of *F. velutipes*.

### 3.4. Soluble Solid Content and Vitamin C

**Figure 3:**
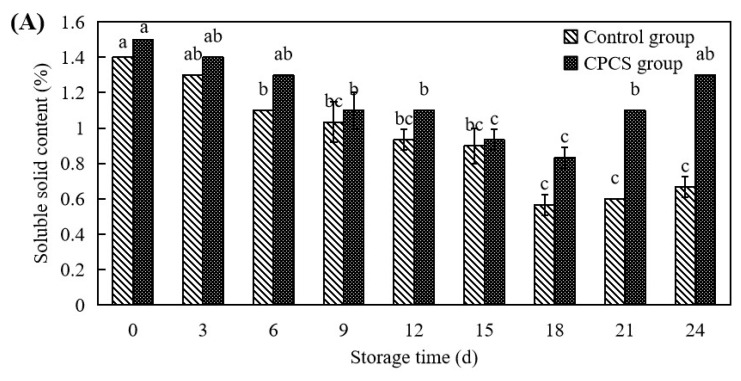

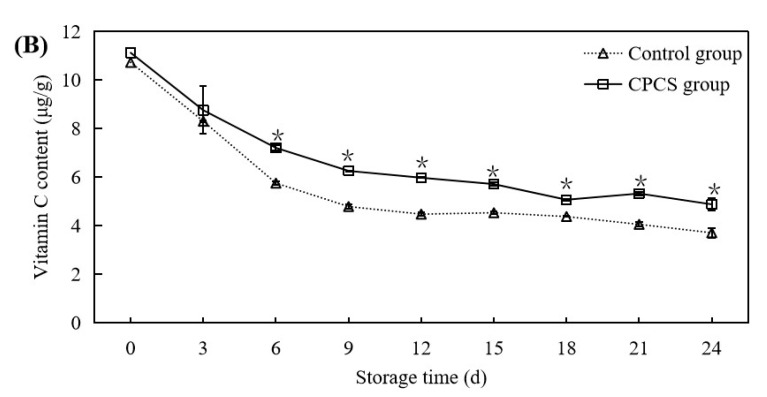
Effects of CPCS on soluble solid (**A**) and vitamin C contents (**B**) of *F. velutipes* during cold storage. ‘*’ indicated the significant differences (*p* < 0.05) between two treatments on each sampling day.

After harvesting, *F. velutipes* constantly consumes its own stored nutrients, and the consumption of sugars provides energy for the respiration of *F. velutipes*. However, the degradation of carbohydrates promotes fibrous deterioration of substrates and causes deterioration of the flavor of *F. velutipes*. Vitamin C is an important nutrient in mushrooms, and in general, the vitamin content of *F. velutipes* decreases with the storage time [23]. The soluble solid content and the vitamin C content are indicators in *F. velutipes* that reflect changes in its nutrients. The soluble solid contents of the two treatments showed a decreasing trend first and then an increasing trend, as the soluble solid content of the CPCS group was higher than that of the control group (Figure 3A). On the 18th day of storage, the soluble solid contents of both groups decreased to the lowest level, which were 0.57 ± 0.06% in the control and 0.83 ± 0.05% in the CPCS group. The soluble solid contents in the two groups then gradually increased, with the CPCS group’s content being double that of the control group at the end of the storage period. During storage, the rate of change in the vitamin C content in the control group was faster than that of the CPCS group (Figure 3B). During the 6th to 12th days of storage, the vitamin C content in the CPCS group was significantly higher than that of the control group (*p* < 0.05). In summary, CPCS-treated *F. velutipes* can effectively delay the loss of soluble solids and vitamin C. However, the mechanisms affecting the elevated soluble solid content of *F. velutipes* in the later stages of storage need to be further investigated in subsequent studies.

### 3.5. Total Phenolic Content, MDA Content, PPO Enzyme Activity, and Degree of Browning

**Figure 4:**
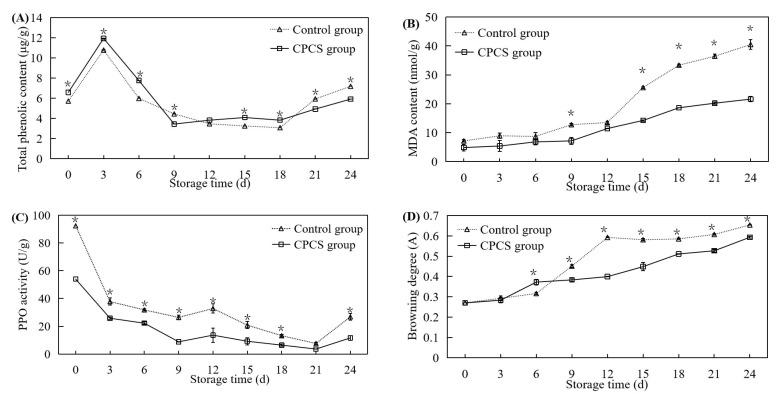
Effects of CPCS on total phenolic (**A**), MDA contents (**B**), PPO enzyme activity (**C**), and browning degree (**D**) of *F. velutipes* during cold storage. ‘*’ indicated the significant differences (*p* < 0.05) between two treatments on each sampling day.

The integrity of cell membranes is important for ensuring the proper functioning of an organism’s physiological activities. When the dynamics of free radical production and scavenging are disrupted within an organism’s cells, the accumulation of ROS can trigger or exacerbate membrane ester peroxidation, causing damage to cell membranes or cell death. The end product of membrane lipid peroxidation is MDA, and MDA accumulation can inversely inhibit cellular antioxidant enzyme activity and reduce antioxidant levels, thereby exacerbating membrane lipid peroxidation. In addition, the browning of higher plant tissues is thought to result from the action of PPO. PPO catalyzes the oxidation of polyphenols to quinones, which polymerize and react with amino acids in intracellular proteins, resulting in melanin deposition. Browning is a sign of deterioration of the quality of *F. velutipes* [24]. These experimental indicators are related to the degree of damage to tissue structure and browning reactions of *F. velutipes* [25]. In the early stages of storage, the total phenolic content of the experimental group was higher than that of the control group, whereas in the later stages, the total phenolic content of the control group was higher than that of the experimental group (Figure 4A). On the 24th day of storage, the total phenolic content of the experimental group was 5.91 ± 0.18 µg/g and that of the control group was 7.18 ± 0.11 μg/g, which was 1.21 times that of the experimental group. The MDA contents of the control group and CPCS groups increased with the increasing storage time (Figure 4B). At the beginning of storage, the MDA contents of the control and CPCS groups were 7.16 ± 0.38 nmol/g and 4.87 ± 1.13 nmol/g, respectively. From the 12th day of storage, the rate of change in MDA content in the control group was significantly higher than that of the CPCS group. On the 24th day of storage, the MDA content of the control group was significantly higher than that of the CPCS group (*p* < 0.05), which was 1.87 times that of the CPCS group.

With an increase in storage time, the changes in PPO activity of *F. velutipes* in the control and CPCS groups showed a downward trend, while the degree of browning showed an upward trend (Figure 4C,D). The PPO activity of the control group was remarkably higher than that of the experimental group during the entire storage period. After 24 days of storage, the PPO enzyme activity of the control group was 26.80 ± 1.70 U/g, which was significantly higher than that of the CPCS group (11.60 ± 2.39 U/g). From the 9th day of storage, the degree of browning in the control group was higher than that of the CPCS group. On the 12th to 15th days of storage, the degree of browning in the control group was significantly higher than that of the experimental group (*p* < 0.05), which is consistent with the color evaluation results.

### 3.6. Antioxidant Enzyme Activity and Free Radical Scavenging Ability

**Figure 5:**
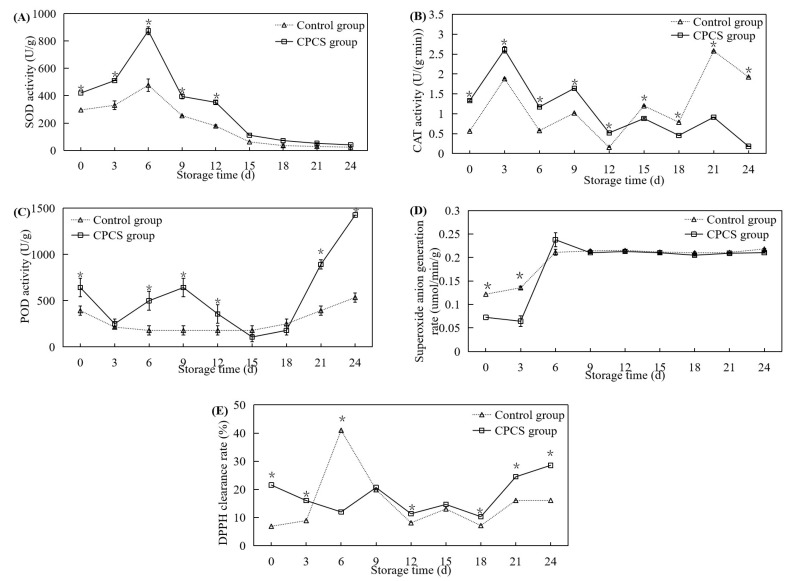
Effects of CPCS on antioxidant enzyme activities ((**A**) SOD; (**B**) CAT; (**C**) POD), superoxide anion generation (**D**), and DPPH clearance rates (**E**) of *F. velutipes* during cold storage. ‘*’ indicated the significant differences (*p* < 0.05) between two treatments on each sampling day.

SOD, CAT, and POD are all important antioxidant enzymes in plants that can effectively scavenge free radicals and achieve the purpose of delaying the aging of tissue cells [26]. With an increase in storage time, the changes in SOD activity in the control and CPCS groups showed a first increasing and then decreasing trend (Figure 5A). During storage, the SOD activity of the CPCS group was significantly higher than that of the control group (*p* < 0.05), and the activities of both groups peaked on the 6th day. At this time, the SOD activities of the control and CPCS groups were 477.26 ± 46.11 U/g and 874.64 ± 28.23 U/g, respectively. During days 0–3, CAT activities in both groups showed an upward trend (Figure 5B). The first peak was on the 3rd day, when the CAT activity of the CPCS group was 1.39 times that of the control group. From the 15th day of storage, CAT activity in the control group was higher than that of the CPCS group. At the later stage of storage, the activity of the control group was higher than that of the CPCS group, and the maximum value in the control group was 2.58 ± 0.01 U/min/g, which was 2.83 times that of the CPCS group. With the prolonged storage time, the changes in POD activity in the control group showed a first slowly decreasing and then increasing trend. On the 15th and 18th days of storage, the POD activity in the control group was higher than that of the CPCS group (Figure 5C). POD activity showed an upward trend, and POD activity in the CPCS group was significantly higher than that of the control group on the 6th, 9th, 12th, 21st, and 24th days of storage (*p* < 0.05).

Superoxide anion radicals are generated by the single-electron reduction of molecular oxygen, which can initiate a chain reaction of free radicals that can convert to oxygen radicals, such as H_2_O_2_, and damage tissue. Superoxide anion radicals are mainly scavenged by superoxide dismutase in the body, and the scavenging rate of superoxide anions can evaluate the scavenging effect of the body; the higher the scavenging rate, the stronger the superoxide dismutase activity and the stronger the antioxidant effect on the plant body [24]. The superoxide anion generation rates in both groups showed an increase first and then stabilized in the range of 0.2 ± 0.1 μmol/min/g (Figure 5D). On the 6th day of storage, the superoxide anion generation rate of the experimental group reached a maximum value of 0.24 ± 0.01 μmol/min/g, which was 1.13 times that of the control group, and the difference was not significant (*p* > 0.05). During the storage period of 0−6 days, the DPPH clearance rate of the control group showed an upward trend reaching the first peak (41%) on the 6th day, which was 3.42 times that of the experimental group. From the 18th day of storage, the changes in DPPH clearance in the control and experimental groups showed an upward trend (Figure 5E). During 12–24 days of storage, the DPPH clearance rates of the CPCS group were significantly higher than those of the control group (*p* < 0.05).

### 3.7. TEM Observation

**Figure 6:**
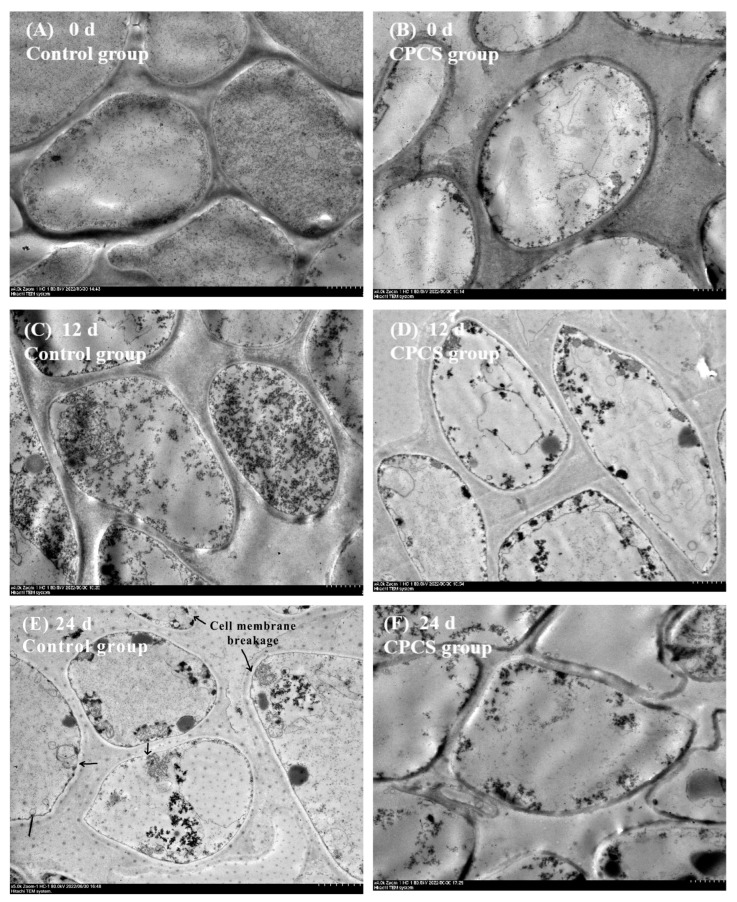
Micromorphological changes in *F. velutipes* cells in the control group (**left**) and the CPCS group (**right**) during cold storage.

*F. velutipes* in the control and CPCS groups was observed by TEM after storage for 0, 12, and 24 days, and the results are shown in Figure 6. At the beginning of storage, mushroom cells in two group exhibited a plump and rounded cellular morphology. Cells in the control group gradually became thinner with the increasing storage time, and some cell membrane structures breakage occurred at the end of storage. Figure 6B,D,F shows the cell structures of *F. velutipes* in the CPCS group stored for 0, 12, and 24 days, respectively. The cells remained relatively plump with the extension of the storage time, and the structure was relatively complete.

## 4. Discussion

Plasma treatment is widely recognized as an effective technique for decontaminating contact surfaces [9]. *F. velutipes* has a fast respiration rate and is very susceptible to microbial contamination; therefore, it is highly perishable after picking, and decontamination with ordinary water is not feasible [27]. This study showed that the total number of colonies in the CPCS group was much lower than that of the control group after cold plasma treatment, indicating that cold plasma treatment could effectively inhibit the growth and multiplication of microorganisms and that a higher treatment voltage and a longer treatment time were more effective for microbial inactivation [28]. The growth rate of the total number of *F. velutipes* colonies fluctuated due to factors including the environment, nutrients, and antagonistic effects. At the same time, cold plasma treatment had no obvious effect on the fresh weight of *F. velutipes*. After 24 days of storage, the rate of weight loss was maintained below 1.2% (Figure 2A). This could be attributed to the fact that mushrooms are only protected by a thin epidermal structure that does not prevent rapid superficial dehydration [29]. By inhibiting PPO enzyme activity, the oxidative browning of F. velutipes was effectively controlled, and the color of *F. velutipes* in the CPCS group was less than that of the control group, which effectively delayed the postharvest morphological changes in *F. velutipes*.

During storage, the oxygen content in the packaging box gradually decreased due to plant respiration. Some studies have shown that the malondialdehyde content and the superoxide anion generation rate of *F. velutipes* increased under anaerobic conditions or modified atmosphere packaging containing 20–50% oxygen. These conditions activated the antioxidant enzyme system, where superoxide dismutase, catalase, and other activities were increased, thereby removing active oxidative substances which resulted in reduced damage during storage [3]. After cold plasma treatment, the MDA content of *F. velutipes* in the CPCS group was lower than that of the control group. At the later stages of storage (15–24 days), the MDA content in the CPCS group was significantly lower than that of the control group (*p* < 0.05) (Figure 4B). Moreover, electron microscopy showed that the cell structure of the CPCS group remained relatively intact, while the cell membrane of the control group was partially broken and the cell contents were incomplete, indicating that cold plasma treatment could effectively reduce the accumulation of MDA in *F. velutipes*, reduce the degree of membrane lipid peroxidation and maintain cell membrane integrity. In addition, during cold plasma generation, many organic compounds can be directly degraded due to the generation of light emission, shock waves, and free radicals [30], and CPCS treatment can increase the content of phenolic compounds in wine [31]. In the early stages of storage, the total phenolic content of the CPCS group was higher than that of the control group; however, on the 24th day of storage, the total phenolic content of the control group was 1.215 times that of the CPCS group (Figure 4A). CPCS treatment can change the cellular respiratory pathway and reduce cell viability, resulting in a decrease in the respiratory rate and heat production of fruits after treatment. At the same time, it can promote the transient stress response in plant tissues and may have adverse effects on nutrients such as vitamin C, anthocyanins, and phenols [32]. Under the treatment conditions of this study, CPCS could effectively delay the decrease in vitamin C content in *F. velutipes* after harvest. During the storage period, the vitamin C content in the CPCS group was higher than that of the control group. On the 24th day of storage, the vitamin C content of the CPCS group was 1.316 times higher than that of the control group (Figure 3B). Some studies have shown that moderate cold plasma treatment of cherries could prolong their shelf life without affecting the contents of soluble solid, total phenols, flavonoids, anthocyanins, and vitamin C [33].

Postharvest storage can be considered abiotic stress for mushrooms, because the storage conditions are very different from the growth conditions. Mechanical damage occurs during picking and transport, which can lead to the inhibition of electron transport in the mitochondria, and the degradation yield of ROS increases [23]. Oxygen-free radical production is further exacerbated during environmental stress; therefore, SOD is thought to be important for stress tolerance in plants [25]. Accumulation of ROS due to an altered balance between ROS production and scavenging capacity will reduce quality during storage and, consequently, the commercial value of fruits and vegetables [34]. This study showed that cold plasma sterilization could induce an increase in the activities of SOD, CAT, and POD; however, CAT enzyme activity at later storage was significantly lower than that of the control group (*p* < 0.05) (Figure 5B). Some studies have shown that plant senescence can lead to changes in the oxidative metabolism of peroxisomes, SOD isoenzymes, and the ascorbate glutathione cycle, as well as changes in the quantity and quality of peroxisomes. Among them, metabolic changes in peroxisome ROS are manifested as the reduction or disappearance of catalase activity and the excessive production of O_2_ and H_2_O_2_. Peroxisomes can also act as a source of ROS transduction signals, causing changes in the antioxidant system [26]. Cold plasma sterilization technology could effectively reduce free radical oxidation damage, delay the quality deterioration of *F. velutipes* and enhance its storage stability, thereby prolonging the shelf life of mushrooms.

## 5. Conclusions

This study demonstrated that CPCS treatment had a positive impact on the storage quality of *F. velutipes*. The CPCS treatment effectively reduced the accumulation of MDA and loss of vitamin C in postharvest *F. velutipes*, alleviated membrane lipid peroxidation and improved the integrity of mushroom cell membranes, thus delaying spoilage and prolonging the shelf life. In addition, cold plasma technology was highly efficient and produced no harmful residual substances. Therefore, this study provides a strong basis for the promotion and application of CPCS technology for the storage, transportation, and preservation of fresh mushrooms in the future. However, the mechanism of the effect of CPCS on the enzyme activity of *F. velutipes* requires further study.

## Figures and Tables

**Table 1:** Changes in microbial growth and sterilization rate of differently treated *Flammulina velutipes* during cold storage *.

	Storage Time (d)								
	0	3	6	9	12	15	18	21	24
Control group (×10^7^ CFU/g)	0.035 ± 0.003 ^f^	0.189 ± 0.049 ^e^	0.192 ± 0.015 ^e^	0.475 ± 0.021 ^d^	0.720 ± 0.098 ^d^	4.245 ± 0.304 ^c^	4.650 ± 0.304 ^c^	8.150 ± 0.636 ^b^	11.000 ± 1.414 ^a^
CPCS group (×10^5^ CFU/g)	0.004 ± 0.002 ^e^	0.009 ± 0.004 ^e^	0.013 ± 0.010 ^e^	0.122 ± 0.009 ^d^	0.172 ± 0.008 ^c^	0.866 ± 0.024 ^b^	1.040 ± 1.130 ^a,b^	1.900 ± 1.830 ^a^	3.950 ± 1.950 ^a^
Sterilization rate (%)	99.88 ^b^	99.95 ^a^	99.93 ^a^	99.74 ^c^	99.76 ^c^	99.79 ^c^	99.78 ^c^	99.77 ^c^	99.64 ^d^

* Different lowercase letters (^a^ to ^f^) for each row of data represent significant differences (*p* < 0.05).

## Data Availability

Not applicable.

## Supplementary Materials

The following supporting information can be downloaded at: https://www.mdpi.com/article/10.3390/life13010070/s1, Figure S1: Effects of CPCS treatment voltage, processing time, processing frequency on the sterilization rate; Table S1: Regression model establishment and significance analysis; Table S2: Analysis of variance results.

## References

[1] Wang P.M., Liu X.B., Dai Y.C., Egon H., Kari S., Yang Z.L. (2018). Phylogeny and species delimitation of *Flammulina*: Taxonomic status of winter mushroom in East Asia and a new European species identified using an integrated approach. Mycol. Prog..

[2] Fang D.L., Wang H.T., Deng Z.L., Benard M.K., Pei F., Hu Q.H., Ma N. (2022). Nanocomposite packaging regulates energy metabolism of mushrooms (*Flammulina filiformis*) during cold storage: A study on mitochondrial proteomics. Postharvest Biol. Technol..

[3] Wang C.T., Wang C.T., Cao Y.P., Nout M.J.R., Sun B.G., Liu L. (2011). Effect of modified atmosphere packaging (MAP) with low and super atmospheric oxygen on the quality and antioxidant enzyme system of golden needle mushrooms (*Flammulina velutipes*) during postharvest storage. Eur. Food Res. Technol..

[4] Xia R.R., Wang L., Xin G., Bao X.J., Sun L.B., Xu H.R., Hou Z.S. (2021). Preharvest and postharvest applications of 1-MCP affect umami taste and aroma profiles of mushrooms (*Flammulina velutipes*). LWT-Food Sci. Technol..

[5] Fang D.L., Yang W.J., Kimatu B.M., Mariga A.M., Zhao L.Y., An X.X., Hu Q.H. (2016). Effect of nanocomposite-based packaging on storage stability of mushrooms (*Flammulina velutipes*). Innovative Food Sci. Emerging Technol..

[6] Xu L.N., Cao W.H., Li R., Zhang H.J., Xia N., Li T., Liu X.X., Zhao X.T. (2019). Properties of soy protein isolate/nano-silica films and their applications in the preservation of *Flammulina velutipes*. J. Food Process. Preserv..

[7] PraveenK M., Pious C.V., Sabu T., Yves G., Thomas S., Mozetič M., Cvelbar U., Špatenka P., Praveen K.M. (2019). Relevance of plasma processing on polymeric materials and interfaces. Non-Thermal Plasma Technology for Polymeric Materials: Applications in Composites, Nanostructured Materials, and Biomedical Fields.

[8] Dimitrakellis P., Gogolides E. (2018). Atmospheric plasma etching of polymers: A palette of applications in cleaning/ashing, pattern formation, nanotexturing and superhydrophobic surface fabrication. Microelectron.

[9] Domonkos M., Ticha P., Trejbal J., Demo P. (2021). Applications of cold atmospheric pressure plasma technology in medicine, agriculture and food industry. Appl. Sci..

[10] Thirumdas R., Sarangapani C., Annapure U.S. (2015). Cold plasma: A novel non-thermal technology for food processing. Food Biophysics.

[11] Pankaj S.K., Bueno-Ferrer C., Misra N.N., Milosavljevic V., O’Donnell C.P., Bourke P., Keener K.M., Cullen P.J. (2014). Applications of cold plasma technology in food packaging. Trends Food Sci. Technol..

[12] Ali M., Cheng J.H., Sun D.W. (2021). Effect of plasma activated water and buffer solution on fungicide degradation from tomato (*Solanum lycopersicum*) fruit. Food Chem..

[13] Butscher D., Van Loon H., Waskow A., von Rohr P.R., Schuppler M. (2016). Plasma inactivation of microorganisms on sprout seeds in a dielectric barrier discharge. Int. J. Food Microbiol..

[14] Sadhu S., Thirumdas R., Deshmukh R.R., Annapure U.S. (2017). Influence of cold plasma on the enzymatic activity in germinating mung beans (*Vigna radiate*). LWT-Food Sci. Technol..

[15] Lacombe A., Niemira B.A., Gurtler J.B., Fan X.T., Sites J., Boyd G., Chen H.Q. (2015). Atmospheric cold plasma inactivation of aerobic microorganisms on blueberries and effects on quality attributes. Food Microbiol..

[16] Lee T., Puligundla P., Mok C. (2018). Intermittent corona discharge plasma jet for improving tomato quality. J. Food Eng..

[17] Fang D.L., Wang C.F., Deng Z.L., Ma N., Hu Q.H., Zhao L.Y. (2021). Microflora and umami alterations of different packaging material preserved mushroom (*Flammulina filiformis*) during cold storage. Food Res. Int..

[18] Fang D.L., Zheng Z.M., Ma N., Yang W.J., Dai C., Zhao M.W., Deng Z.L., Hu Q.H., Zhao L.Y. (2020). Label-free proteomic quantification of packaged *Flammulina filiformis* during commercial storage. Postharvest Biol. Technol..

[19] Bristy A.T., Islam T., Ahmed R., Hossain J., Reza H.M., Jain P. (2022). Evaluation of total phenolic content, HPLC analysis, and antioxidant potential of three local varieties of mushroom: A comparative study. Int J Food Sci..

[20] Zhao M., Zhang Z., Cai H., Wang L., Hu C., Li D., Chen Y., Kang Y., Li L. (2022). Controlled moisture permeability of thermoplastic starch/polylactic acid/poly butylene adipate-co-terephthalate film for the autolysis of straw mushroom *Volvariella volvacea*. Food Chem..

[21] Ghosh S., Nandi S., Banerjee A., Sarkar S., Chakraborty N., Acharya K. (2021). Prospecting medicinal properties of Lion’s mane mushroom. J. Food Biochem..

[22] Li P., Zhang X., Hu H., Sun Y., Wang Y., Zhao Y. (2013). High carbon dioxide and low oxygen storage effects on reactive oxygen species metabolism in *Pleurotus eryngii*. Postharvest. Biol. Technol..

[23] Wang X.M., Zhang J., Wu L.H., Zhao Y.L., Li T., Li J.Q., Wang Y.Z., Liu H.G. (2014). A mini-review of chemical composition and nutritional value of edible wild-grown mushroom from China. Food Chem..

[24] Yang W.S., Stockwell B.R. (2016). Ferroptosis: Death by lipid peroxidation. Trends Cell Biol..

[25] Chris B., Wim V.C., Marc V.M., Dirk I., Professor K.A. (1994). Superoxide dismutase in plants. Crit. Rev. Plant Sci..

[26] del Rio L.A., Pastori G.M., Palma J.M., Sandalio L.M., Sevilla F., Corpas F.J., Jimenez A., Lopez-Huertas E., Hernandez J.A. (1998). The activated oxygen role of peroxisomes in senescence. Plant Physiol..

[27] Nguyen P.M. (2022). Corona discharge power of plasma treatment influence on the physicochemical and microbial quality of enoki mushroom (*Flammulina velutipes*). J. Pure Appl. Microbiol..

[28] Bermudez-Aguirre D., Wemlinger E., Pedrow P., Barbosa-Canovas G., Garcia-Perez M. (2013). Effect of atmospheric pressure cold plasma (APCP) on the inactivation of *Escherichia coli* in fresh produce. Food Control.

[29] Jiang T. (2013). Effect of alginate coating on physicochemical and sensory qualities of button mushrooms (*Agaricus bisporus*) under a high oxygen modified atmosphere. Postharvest Biol. Technol..

[30] He F., Liang N.N., Mu L., Pan Q.H., Wang J., Reeves M.J., Duan C.Q. (2012). Anthocyanins and their variation in red wines I. Monomeric anthocyanins and their color expression. Molecules.

[31] Niedzwiedz I., Plotka-Wasylka J., Kapusta I., Simeonov V., Stoj A., Wasko A., Pawlat J., Polak-Berecka M. (2022). The impact of cold plasma on the phenolic composition and biogenic amine content of red wine. Food Chem..

[32] Pan Y.W., Cheng J.H., Sun D.W. (2020). Inhibition of fruit softening by cold plasma treatments: Affecting factors and applications. Crit. Rev. Food Sci. Nutr..

[33] Wu X.R., Zhao W.Q., Zeng X.Y., Zhang Q.A., Gao G.T., Song S.J. (2020). Effects of cold plasma treatment on cherry quality during storage. Food Sci. Technol. Int..

[34] Hodges D.M., Lester G.E., Munro K.D., Toivonen P.M. (2004). Oxidative stress: Importance for postharvest quality. Hortscience.

