# Supplementary material for: Storage Quality Variation of Mushrooms (Flammulina velutipes) after Cold Plasma Treatment
Source: Life (Basel). 2022 Dec 26;13(1):70. doi: 10.3390/life13010070 (PMC9864181; doi:10.3390/life13010070)
Supplement: Supplementary file 1 [file life-13-00070-s001.zip › life-2084982-supplementary.pdf]

1. Processing conditions optimization of cold plasma treatment

1.1 Single-factor experiment

F. velutipes (50 g) was randomly packaged in a polypropylene packaging box (17×12×3.2 cm) and sealed, and each treatment group was repeated three times. The dielectric barrier discharge (DBD) cold plasma conditions included air as the working gas, the treatment time, treatment voltage, and treatment frequency of the air at a temperature of 4 °C, relative humidity of 90%, and a distance between the plates of 29 cm. Taking the *F. velutipes* without plasma treatment as the control, the microbial indicators (total number of microbial colonies) of *F. velutipes* were measured respectively, and each index was measured three times.

1.2 Single-factor test

(1) Effect of CPCS treatment frequency on the sterilization rate of *F. velutipes*: the fixed treatment voltage was 75 kV, the treatment time was 180 s, the number of repeated treatments was 1, and the treatment frequencies were 60, 90, 120, 150, 180, and 200 Hz.

(2) Effect of CPCS treatment voltage on the sterilization rate of *F. velutipes*: the fixed treatment frequency was 120 Hz, the treatment time was 180s, the number of repeated treatments was 1, and the treatment voltages were 55, 65, 75, 85, 95, and 105 kV.

(3) Effect of CPCS treatment time on the sterilization rate of *F. velutipes*: the fixed treatment voltage was 75 kV, the treatment frequency was 120 Hz, the number of repeated treatment times was 1 time, and treatment times were 60, 90, 120, 150,

180, and 210 s.

1.3 Response surface method optimization

Based on the single-factor test results, taking the treatment voltage, treatment frequency, and treatment time as independent variables, the sterilization rate was the response value, and the response surface optimization of the sterilization rate of *F. velutipes* was carried out.

2. Results

2.1 Single-factor experiment

2.1.1 Effect of CPCS treatment voltage on sterilization rate

The sterilization rate also increased with an increase in processing voltage. When the voltage treatment conditions were 85, 95, and 105 kV, the sterilization rate was greater than 80%, and the sterilization effect was relatively stable. Therefore, the selected voltages for the subsequent response surface tests were 85 kV, 95 kV, and 105 kV (Fig. S1A).

2.1.2 Effect of CPCS processing time on sterilization rate

The sterilization rate gradually increased with increasing treatment time, and the sterilization rate changed significantly when the treatment time was between 60-150 s. When the treatment times were 150, 180, and 210 s, the sterilization rate was more than 80%, and the data error was small and stable under the same conditions. The overall sterilization effect showed an increasing trend over time. Therefore, the selected times for the follow-up response surface test were 150, 180, and 210 s (Fig.

S1B).

2.1.3 Effect of CPCS processing frequency on sterilization rate

The sterilization rate also increased with an increase in processing frequency. When the frequency treatment conditions were 150, 180, and 200 Hz, the sterilization rates were 87.5, 85.9, and 87.3%, respectively, and the data were relatively stable under the same conditions. Therefore, the frequencies selected for the subsequent response surface tests were 150, 180, and 210 Hz (Fig. S1C).

2.2 Response surface method optimization

2.2.1 Response surface factor level design

According to the single-factor test results, a three-factor three-level response surface optimization test was designed with processing voltage, processing frequency, and processing time as the influencing factors and sterilization rate as the index (Table 1).

2.2.2 Regression model establishment and significance analysis

Design-Expert software was used to carry out quadratic polynomial regression fitting analysis on the test data, and the regression equation of the sterilization rate on independent variables was obtained as follows:

$$Y=83.75-2.58A-0.7130B-0.2884C+0.3795AB+0.2382AC+0.7565BC-5.08A^2+13.71B^2+0.2244C^2$$

As shown in table 2, the regression model is significant ($P<0.0001$), and the mismatch is not significant ($P=0.0722>0.05$), indicating that the model fits the test well. $R^2=0.8506>0.8$, which effectively reflects the relationship between the

sterilization effect of low-temperature plasma and various treatment factors. B^2 (processing time × processing time) significantly affects the sterilization rate.

According to the analysis of Design-Expert software, the best process conditions were a treatment frequency of 151.631 Hz, a treatment voltage of 94.308 kV, a treatment time of 150.102 s, and a sterilization rate of 99.440%. Considering the requirements of the actual equipment parameter settings, the optimal process conditions were adjusted as follows: treatment frequency of 150 Hz, treatment voltage of 95 kV, and treatment time of 150 s. After correction, the sterilization rate was 99.439% (n=3), and the relative error was 0.10%. The results indicated that the predicted value of the model was in good agreement with the actual test value, and the optimization result of the response surface methodology was reliable.

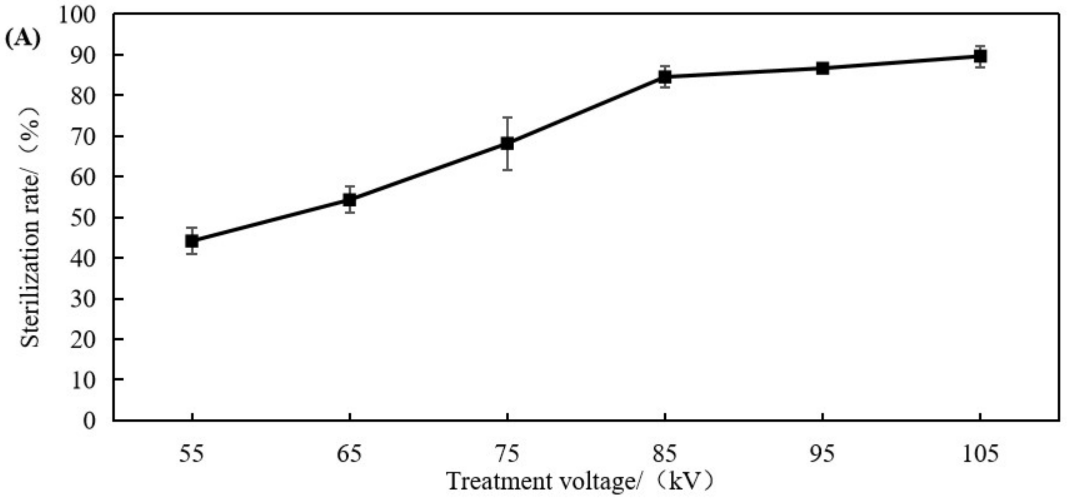

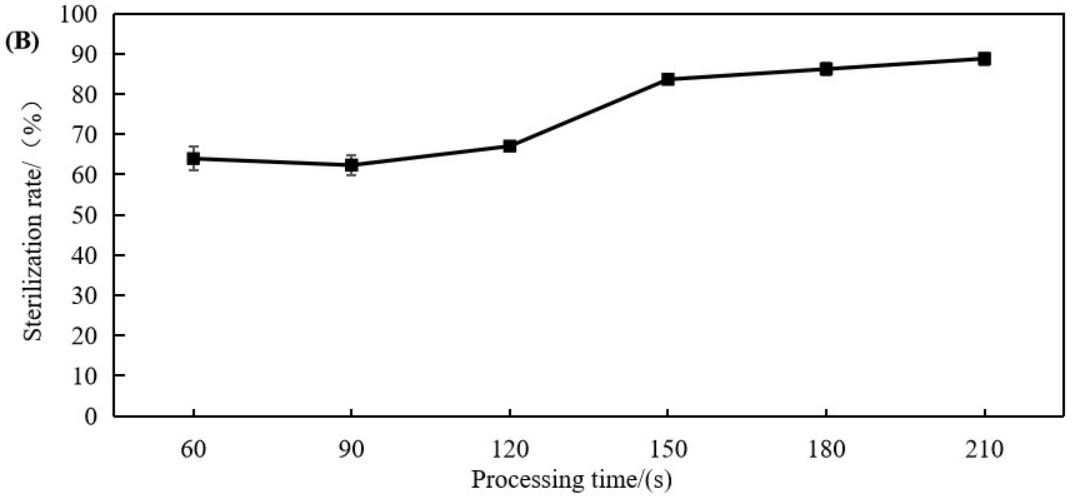

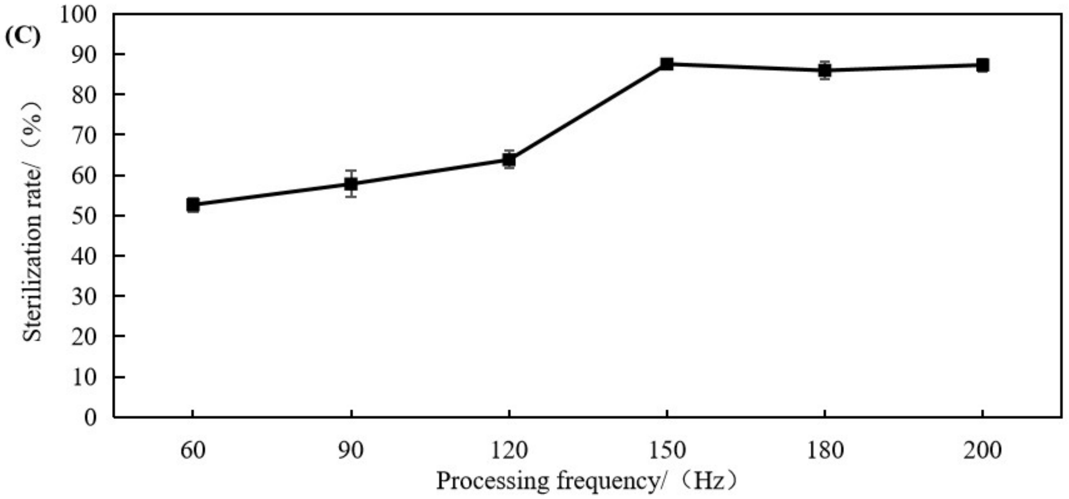

Figure S1 Effect of CPCS treatment voltage, processing time, processing frequency on sterilization rate

Table S1 Regression model establishment and significance analysis

Serial number	A(Treatment voltage）	B（Processing time）	C（Processing frequency）	Sterilization rate
1	95	180	180	83.5
2	95	210	150	95.2727
3	95	180	180	80.0909
4	85	210	180	98.4121
5	95	210	200	97.45
6	95	180	180	87.9545
7	95	180	180	84.5061
8	105	180	150	80.2121
9	85	180	150	79.6364
10	85	150	180	99.0818
11	95	150	150	99.1818

12	105	150	180	85.4848
13	105	210	180	86.3333
14	95	180	180	82.45
15	95	150	200	98.8182
16	105	180	200	80.1818
17	85	180	200	75.5455

Table S2 Analysis of variance results

Source	Square sum	Freedom	Mean square	F-value	P-value	Significance
Model	933.10	9	103.68	4.43	0.0313	*
A	52.30	1	52.30	2.23	0.1787	
B	3.99	1	3.99	0.1703	0.6922	
C	0.6656	1	0.6656	0.0284	0.8709	

AB	0.5762	1	0.5762	0.0246	0.8798	
AC	0.2315	1	0.2315	0.0099	0.9236	
BC	2.33	1	2.33	0.0997	0.7614	
A^2	108.64	1	108.64	4.64	0.0682	
B^2	791.11	1	791.11	33.78	0.0007	*
C^2	0.1914	1	0.1914	0.0082	0.9305	
Residual	163.93	7	23.42			
Missing item	130.55	3	43.52	5.21	0.0722	
Pure error	33.38	4	8.34			
The sum	1097.03	16				

* $P<0.05$.
